# Supplementary material for: Blood n-3 fatty acid levels and total and cause-specific mortality from 17 prospective studies
Source: Nat Commun. 2021 Apr 22;12:2329. doi: 10.1038/s41467-021-22370-2 (PMC8062567; doi:10.1038/s41467-021-22370-2)

**Supplementary Table 1. Relevant background information by cohort**

|  |  | **60Y0** |
| --- | --- | --- |
| **Cohort** | Full Name | The Swedish 60-year-olds cohort (60YO) |
| **Descriptive + Acknowledgement** | Field centers/Cities, State; Country (Sample source) | Stockholm county, Sweden; |
|  | Description | The 60YO is a population-based cohort including Swedish men and women, aged 60 years at the time of enrollment. From August 1997 to March 1999, every third man and woman who was born between 1 July 1937 and 31 June 1938 (60 years old) and living in Stockholm County, Sweden, was invited to participate in a screening for cardiovascular disease (CVD) risk factors. Among the participants invited (n=5,460), 4,232 (78% response rate), 2,039 men and 2,193 women, agreed to participate. At baseline, participants underwent to a physical examination including anthropometric measurements and blood pressure and completed an extensive questionnaire about their disease history, health status, medication therapy, lifestyle, and nutritional habits. Blood samples were also drawn after overnight fasting. Participants were followed-up for CVD and death till December, 31rst 2017 |
|  | References | Nutr Metab Cardiovasc Dis 2007;17:349–57. |
|  | Contact | Karin Leander (Karin.Leander@ki.se) |
|  | Funding Information | Stockholm County Council, Swedish Heart and Lung-Foundation, Swedish Research Council, The Swedish Research Council for Longitudinal Research, ALF, The Cardiovascular Programme in Stockholm, The Strategic Research in Epidemiology at Karolinska Institute |
| **Ascertainment of Fatty Acid Biomarker Concentrations** | Please provide a brief description of methods used to ascertain fatty acid concentrations, including laboratory CVs | Serum samples, collected at the time of the recruitment (1997-1999), were stored at -80°C until the analyses were performed in 2012. The percentage composition of methylated fatty acids was determined by gas-chromatography(GC) with a ﬂame ionization detector and helium as the carrier gas. To avoid contamination of the GC column, free cholesterol liberated in the reaction was removed using an aluminum oxide column. The gas-liquid chromatography (GLC) system used for the analysis consisted of a 30-m glass capillary column coated with Thermo TR-FAME (Thermo Electron Corporation, Waltham, MA, USA), and an Agilent Technologies system consisting of model GLC 6890N, an autosampler 7683 and Agilent ChemStation (Agilent Technologies Inc., Santa Clara, CA, USA). The temperature was programmed to 150–260°C. Thirteen fatty acids were identiﬁed using standards from Nu Check Prep (Elysian, MN, USA). Individual serum cholesterol fatty acids were expressed as a proportion of the sum of all fatty acids measured. Fatty acid composition in one serum sample was repeatedly analyzed in duplicates in all batches for quality control and the intra- and inter-assay coefficient of variations were ≤0.24 and ≤2.49 %, respectively, for the fatty acids utilized for statistical analyses (ALA, EPA, DHA, LA, and AA). |
| **Outcome Assessment** | Mortality Definitions  And Maximum Follow-up Time for the four major mortality outcomes | All-cause and specific-cause mortality data were obtained through linkage to the Swedish Cause of Death register up to December 31, 2017. The International Classification of Disease 10th Revision (ICD 10th) was used for the identification of the specific-cause mortality as follows: cancer (ICD-10 C00-C97), CVD (ICD-10 I00-I13, I20-51, I160-171), infections (ICD-10 A00-B99), respiratory (ICD-10J00-J99) and dementia/Alzheimer (ICD10 G30-32) |

|  |  | **AGES-R** |
| --- | --- | --- |
| **Cohort** | Full Name | Age, Gene/Environment Susceptibility-Reykjavik study |
| **Descriptive + Acknowledgement** | Field centers/Cities, State; Country (Sample source) | Reykjavik Iceland |
|  | Description | The AGES-Reykjavik Study is a random sample of 5,764 men and women who were drawn from an established single center population based cohort; the Reykjavik Study, begun in 1967 to study heart disease. AGES-Reykjavik Study was designed to examine risk factors, including genetic susceptibility and gene/environment interaction, in relation to disease and disability in old age. At study baseline (2002–2006), participants were aged 66–96 years. A total of 753 adults with available data on circulating fatty acids and diabetes were eligible for the current analysis. |
|  | References | [http://www.ncbi.nlm.nih.gov/pubmed/17351290, Harris TB, Launer LJ, Eiriksdottir G et al. Age, Gene/Environment Susceptibility-Reykjavik study: multidisciplinary applied phenomics. Am J Epidemiol 2007;165(9):1076-87.](http://www.chs-nhlbi.org/%20Ann%20Epidemiol.%201(3):%20263-276,%201991%20Fried,%20L.P.%20et%20al.%20The%20Cardiovascular%20Health%20Study:%20design%20and%20rationale.%20Ann%20Epidemiol%201,%20263-76(1991).) |
|  | Contact | Vilmundur Gudnason (v.gudnason@hjarta.is) |
|  | Acknowledgements | We thank Pho Diep for technical assistance with fatty acid analyses |
|  | Funding Information | Supported by The Office of Dietary Supplements, NIH contract N01-AG012100, the NIA Intramural Research Program, Hjartavernd (the Icelandic Heart Association), the Althingi (the Icelandic Parliament), Canadian Cancer Society (grant #704735) and the Michael Smith Foundation for Health Research (#17644) |
| **Ascertainment of Fatty Acid Biomarker Concentrations** | Please provide a brief description of methods used to ascertain fatty acid concentrations, including laboratory CVs | Blood samples were collected at the AGES-Reykjavik baseline after an overnight fast and stored at -80C. Fatty acids were measured in plasma phospholipids at the Biomarker Laboratory, Fred Hutchinson Cancer Research Center. Plasma lipids were extracted by using the method of Folch. Phospholipids were separated from other lipids by using one-dimensional thin-layer chromatography. Fatty acid methyl esters were prepared by direct transesterification (19) and separated by using gas chromatography (Agilent Technologies 7890 Gas Chromatograph flame ionization detector; Supelco fused silica 100-m capillary column SP-2560; initially at 1608C for 16 min, ramped up at 3.08C/min to 2408C, and held for 15 min). The identification, precision, and accuracy were continuously evaluated by using both model mixtures of known fatty acid methyl esters and established in-house control pools. Fatty acids were expressed as the weight percentage of the total phospholipid fatty acids analyzed. The CV from pooled quality-control samples for LA, AA, ALA, EPA, DHA, and DPA were all ,2.5%. CVs for other major fatty acids were 0.77% (palmitic), 0.47% (stearic), and 0.42% (oleic). Ref: Harris TB, Song X, Reinders I et al. Plasma phospholipid fatty acids and fish-oil consumption in relation to osteoporotic fracture risk in older adults: the Age, Gene/Environment Susceptibility Study. Am J Clin Nutr 2015; 101(5):947-55. |
| **Outcome Assessment** | Mortality Definitions  And Maximum Follow-up Time for the four major mortality outcomes | Mortality was determined from the Icelandic National Roster. Fact and cause of death were obtained from Statistics Iceland which classified cause of death based on a nosologist review of medical and death records. CVD related deaths included ICD 10th revision codes I10-I25, I42-I52, I61, I63-I74 |

|  |  | **CCCC** |
| --- | --- | --- |
| **Cohort** | Full Name | Chin-Shan Community Cardiovascular Cohort Study |
| **Descriptive + Acknowledgement** | Field centers/Cities, State; Country (Sample source) | One Taiwan community; the Chin-Shan County, New Taipei City, Taiwan |
|  | Description | The CCCC Study is a prospective population-based cohort study of people more than or equal to 35 years old at baseline to evaluate the cardiovascular disease occurrence and related risk factors. Participants were recruited at one center during 1990 and 1991 in the Chin-Shan community, New Taipei City in Taiwan, from the samples of community household lists. The cohort consisted of 3602 non-institutional men and women. A total of 1834 adults with available data on circulating fatty acids and free from diabetes in baseline and available follow-up person-year data were eligible for the current analysis |
|  | References | [Lee, Y. T., et al. (2000). "Chin-Shan Community Cardiovascular Cohort in Taiwan: baseline data and five-year follow-up morbidity and mortality." Journal of Clinical Epidemiology 53: 836-846.](http://www.chs-nhlbi.org/%20Ann%20Epidemiol.%201(3):%20263-276,%201991%20Fried,%20L.P.%20et%20al.%20The%20Cardiovascular%20Health%20Study:%20design%20and%20rationale.%20Ann%20Epidemiol%201,%20263-76%20(1991).) |
|  | Contact | Kuo-Liong Chien ([klchien@ntu.edu.tw](mailto:klchien@ntu.edu.tw)) |
|  | Acknowledgements | The authors express their gratitude to the CCCC participants. |
|  | Funding Information | This CCCC Study research was supported by the grants of the National Science Council and the Ministry of Science and Technology, Taiwan (MOST 106-2314-B-002 -158 -MY3, NSC 102-2314-B-002 -080 -MY2, NSC 100-2314-B-002 -113 -MY3, NSC 97-2314-B-002 -130 -MY3 ), National Taiwan University Hospital (NTUH 106-S3453) and National Taiwan University (NTU--101R7622-3). The content is solely the responsibility of the authors and does not necessarily represent the official views of the funding offices. |
| **Ascertainment of Fatty Acid Biomarker Concentrations** | Please provide a brief description of methods used to ascertain fatty acid concentrations, including laboratory CVs | A 10-ml tube of EDTA-anticoagulated blood was collected, refrigerated at the site centers, then the blood was centrifuged at 800 x g for 10 min. The resulting plasma was separated and dispensed into several aliquots and frozen at -70°C for analysis for fatty acid content by the same technician. After thawing, 0.5 mL of plasma was extracted with 0.5 mL methanol, followed by 1.0 mL chloroform under a nitrogen atmosphere for lipid extraction. A 5890 gas chromatograph (Hewlett Packard, Avondale, PA, USA) equipped with a 30m-FFAT WCOT glass capillary column (J & W Scientific, Folsom, CA, USA) and a flame-ionization detector was performed in separated methyl esters, and the 29 individual fatty acid peaks were ascertained by comparing the retention time of each peak relative to the retention times of FAs of synthetic standards of known FA components. The relative amount of each FA (% of total FAs) was quantified by integrating the area under the peak and dividing the result by the total area for all FAs. |
| **Outcome Assessment** | Mortality Definitions  And Maximum Follow-up Time for the four major mortality outcomes | The ascertainment of the outcomes, we used the official death certificate from the government, then checked the death causes from oral interview. The maximum follow-up time for total mortality was 23.8 years. |

|  |  | **CHS** |
| --- | --- | --- |
| **Cohort** | Full Name | Cardiovascular Heart Study (CHS) |
| **Descriptive + Acknowledgement** | Field centers/Cities, State; Country (Sample source) | 4 U.S. communities: Forsyth County, North Carolina; Sacramento County, California; Washington County, Maryland; Allegheny County, Pennsylvania |
|  | Description | The CHS Study is a prospective population-based cohort study of people ≥ 65 years old at baseline initiated to evaluate risk factors for the development and progression of cardiovascular disease. Participants were recruited at four field centers (Forsyth County, NC; Sacramento County, CA; Washington County, MD; Pittsburgh, PA) from random samples of Medicare eligibility lists. The cohort consists of 5201 non-institutionalized men and women, recruited in 1989-1990, plus an additional 687 black participants recruited in 1992-93. A total of 3941 adults with available data on circulating fatty acids were eligible for the current analysis. |
|  | References | [http://www.chs-nhlbi.org/ Ann Epidemiol. 1(3): 263-276, 1991 Fried, L.P. et al. The Cardiovascular Health Study: design and rationale. Ann Epidemiol 1, 263-76 (1991).](http://www.chs-nhlbi.org/%20Ann%20Epidemiol.%201(3):%20263-276,%201991%20Fried,%20L.P.%20et%20al.%20The%20Cardiovascular%20Health%20Study:%20design%20and%20rationale.%20Ann%20Epidemiol%201,%20263-76%20(1991).) |
|  | Contact | Rozenn Lemaitre ([rozenl@uw.edu](mailto:rozenl@uw.edu)) |
|  | Acknowledgements | The authors express their gratitude to the CHS participants. |
|  | Funding Information | This Cardiovascular Heart Study research was supported by NHLBI contracts HHSN268201200036C, HHSN268200800007C, HHSN268201800001C, N01HC55222, N01HC85079, N01HC85080, N01HC85081, N01HC85082, N01HC85083, N01HC85086; and NHLBI grants U01HL080295, R01HL085710 and U01HL130114 with additional contribution from the National Institute of Neurological Disorders and Stroke (NINDS). Additional support was provided through AG023629 from the National Institute on Aging (NIA). A full list of principal CHS investigators and institutions can be found at CHS-NHLBI.org/. The content is solely the responsibility of the authors and does not necessarily represent the official views of the National Institutes of Health. |
| **Ascertainment of Fatty Acid Biomarker Concentrations** | Please provide a brief description of methods used to ascertain fatty acid concentrations, including laboratory CVs | Plasma phospholipid fatty acids were measured at the Fred Hutchinson Cancer Research Center (Seattle, WA) using stored blood samples from 1992-1993 as previously described (Mozaffarian et al, Ann Intern Med 2011;155:160). Total lipids were extracted from plasma using the methods of Folch. A one dimensional thin-layer chromatography was used to separate phospholipids from neutral lipids. Phospholipids fraction was directly trans-esterified using the Lepage and Roy method to prepare fatty acid methyl esters, and individual fatty acid methyl esters were separated using gas chromatography (Agilent 5890 Gas Chromatograph flame ionization detector, Agilent Technologies, Palo Alto, CA; fused silica capillary column SP-2560 [100m x 0.25mm, 0.2μm], Supelco Belefonte, PA; initial 160 degrees Celsius for 16 min, ramp 3 degrees Celsius/min to 240 degrees Celsius, hold 15 minutes). All fatty acids were processed at the Biomarker Laboratory of the Fred Hutchinson Cancer Research Center (Seattle, WA). For this analysis, levels of each individual fatty acid are expressed as a weight percentage of total phospholipid fatty acids analyzed. Inter-assay coefficient of variations for the EPA, DHA and EPA measurements were ≤ 3%. |
| **Outcome Assessment** | Mortality Definitions  And Maximum Follow-up Time for the four major mortality outcomes | CVD events are adjudicated by a centralized CHS events committee based on information from outpatient and inpatient medical records, diagnostic tests and consultations, and interviews. All-cause and cause-specific mortality are adjudicated by the CHS events committee based on available information from interviews or next of kin, death certificates, and review of medical records including diagnostic tests and consultations, as appropriate. For deaths, underlying cause is adjudicated as CVD mortality, which includes CHD, stroke, other atherosclerotic disease, and other CVD. Non-CVD mortality is also adjudicated, including from cancer, pulmonary diseases, infection, dementia, fractures or trauma, and other causes. Mortality has been adjudicated through 2015. |

|  |  | | **CSHA** |
| --- | --- | --- | --- |
| **Cohort** | Full Name | | Canadian Study of Health and Aging |
| **Descriptive + Acknowledgement** | Field centers/Cities, State; Country (Sample source) | | 18 research centers participating; 36 cities nearby the centers; the 10 provinces of Canada; Canada |
|  | Description | | The CSHA is a large national longitudinal study of people aged 65 years and over. It began in 1991–1992, with follow ups in 1996–1997 and in 2001–2002. In 1991–1992, data were collected from representative samples of men and women aged 65 years and over in 36 urban and surrounding rural areas covering all 10 Canadian provinces (1). Of the 10 263 participants, 9008 lived in the community, and 1255 were residents in institutions. We obtained the community sample from the databases of the provincial health insurance plans except in Ontario, where we used the Enumeration Composite Record, an aggregate list based on election and other municipal records. Subjects were randomly selected by computer in the following age groups: 65 to 74 years, 75 to 84 and 85 and over. Since both the size of the population and the expected rates of dementia vary by age, we used an optimal allocation procedure to reduce variance estimates. |
|  | References | | [CMAJ. 1994 Mar 15;150(6):899-913. Canadian study of health and aging: study methods and prevalence of dementia.](http://www.chs-nhlbi.org/%20Ann%20Epidemiol.%201(3):%20263-276,%201991%20Fried,%20L.P.%20et%20al.%20The%20Cardiovascular%20Health%20Study:%20design%20and%20rationale.%20Ann%20Epidemiol%201,%20263-76%20(1991).) |
|  | Contact | | Danielle Laurin (Danielle.Laurin@pha.ulaval.ca) |
|  | Funding Information | | Phases 1 and 2 of the core study were funded by the Seniors’ Independence Research Program, through Health Canada’s National Health Research and Development Program (NHRDP). The Medical Research Council provided supplementary funding for analysis of the caregiver component. Additional funding was provided by Pfizer Canada Incorporated, through the Medical Research Council/Pharmaceutical Manufacturers Association of Canada Health Activity Program, NHRDP; by Bayer Incorporated; and by the British Columbia Health Research Foundation. Core funding for phase 3 was obtained from the Canadian Institutes for Health Research (CIHR). Supplementary funding for the caregiver component was obtained from CIHR. Merck-Frosst and Janssen-Ortho provided additional funding. The CSHA was coordinated through the University of Ottawa and Health Canada. |
| **Ascertainment of Fatty Acid Biomarker Concentrations** | Please provide a brief description of methods used to ascertain fatty acid concentrations, including laboratory CVs | | Whole blood was separated from plasma by centrifugation and stored in liquid nitrogen. All blood samples were analyzed de novo because they came from a different blood compartment than in the exploratory analysis (16). Frozen EDTA (added as an anticoagulant) whole-blood samples were analyzed at the Lipid Research Centre, Centre Hospitalier Universitaire de Quebec Research Center, Quebec, Canada. Erythrocyte membranes were isolated by centrifugation and washed twice with 0.9% sodium chloride solution. The purified membranes were spiked, with phosphatidylcholine C:15 used as internal standard. Total lipids were extracted by using a mixture of chloroform-methanol (2:1, vol:vol) according to a modified Folch method, and phospholipids were isolated by thin-layer chromatography (33). Phospholipid fatty acids were then methylated (34), and fatty acid profiles were obtained by capillary gas chromatography with an HP5890 gas chromatograph (Hewlett-Packard, Toronto, Canada) equipped with an HP-88 capillary column (100 m · 0.25 mm internal diameter · 0.20 mm film thickness; Agilent Technologies, Santa Clara, CA) coupled with a flame ionization detector. Helium was used as carrier gas (split ratio: 1:80). Fatty acids were identified according to their retention time with a standard mixture of 43 methylated fatty acids (Supelco Inc, Bellefonte, PA). Results are expressed as a percentage of total fatty acids. The detection limit was 0.1%, and the intraassay CV ranged from 0.87% to 4.4% for total n23 fatty acids, from 1.4% to 6.1% for EPA and from 1.3% to 6.8% for DHA. |
| **Outcome Assessment** | Mortality Definitions  And Maximum Follow-up Time for the four major mortality outcomes | | For participants who died before the first follow-up study, and also for those who died between the first and second follow-up, the date and cause of death were obtained from the Provincial Registrar of Vital Statistics, and a relative or other informant was interviewed to assess the subject's physical and cognitive status three months prior to death. Unfortunately there is only partial coding of cause of death since our focus was on dementia and it is rarely coded as the underlying cause of death; therefore, the partially available cause of death information has been made available. The maximum follow-up time for all mortality outcomes was 10.3 years. |
|  |  | |  |
|  | |  | **EPIC-Norfolk** |
| **Cohort** | | Full Name | The European Prospective Investigation into Cancer (EPIC)-Norfolk |
| **Descriptive + Acknowledgement** | | Field centers/Cities, State; Country (Sample source) | Norfolk, England |
|  |  | Description | The European Prospective Investigation into Cancer (EPIC)-Norfolk is a prospective study of 25,639 men and women aged 40–79 years in Norfolk, UK similar in characteristics to UK general population samples, who participated in a baseline survey in 1993–1997. Participants completed a health and lifestyle questionnaire including data on medical history, smoking, alcohol intake, physical activity, social class, and education and attended a clinic for a health examination. EPIC-Norfolk participants were not asked to fast before giving blood. Blood samples were spun, separated into 0.5 ml fractions of serum and citrated plasma, placed in straws, sealed, and stored in liquid nitrogen. |
|  |  | References | [DOI 10.22025/2019.10.105.00004, and 10.1371/journal.pmed.1001255](https://journals.plos.org/plosmedicine/article?id=10.1371/journal.pmed.1001255) |
|  |  | Contact | Kay-Tee Khaw ([kk101@cam.ac.uk](mailto:kk101@cam.ac.uk)) |
|  |  | Acknowledgements | We thank Angela Mulligan and Connie Tang for support with data processing and data management. |
|  |  | Funding Information | The EPIC Norfolk study (DOI 10.22025/2019.10.105.00004) has received funding from the Medical Research Council (MR/N003284/1 and MC-UU_12015/1) and Cancer Research UK (C864/A14136). NJW, NGF, and FI were supported by the Medical Research Council Epidemiology Unit core funding [MC_UU_12015/1 and MC_UU_12015/5]. NJW and NGF acknowledge support from the National Institute for Health Research Cambridge Biomedical Research Centre [IS-BRC-1215-20014] and NJW is an NIHR Senior Investigator. |
| **Ascertainment of Fatty Acid Biomarker Concentrations** | | Please provide a brief description of methods used to ascertain fatty acid concentrations, including laboratory CVs | Citrated plasma straws were retrieved from liquid nitrogen storage, thawed at room temperature and 20 mg of di-palmitoyl-D31-phosphatidylcholine (Sigma, St. Louis, MO) internal standard was added to each 200 ml plasma sample. Following extraction of total lipids with chloroform/methanol, phospholipids were further purified by adsorption chromatography (LC-Si SPE, Supelco/Sigma, St. Louis, MO), transmethylated to fatty acid methyl esters and extracted with hexane. Analysis was carried out by gas chromatography with flame ionisation detection (220°C) using a 30 m x 0.32 mm x 0.2 um SP2340 fused silica capillary column (Supelco/Sigma, St. Louis, MO). Carrier gas was Helium at a constant flow of 1.3 ml/min. Samples of 0.5 µl were introduced onto the column via on-column injection. The column was held initially for 1 min at 65°C, then programmed at 5°C/min to 135°C, then at 2°C/min to 200°C, and finally at 10°C/min to 220°C. Run time was 60 min.   Identification of individual fatty acid methyl esters was based on comparison with retention times of authentic standards (Sigma, St. Louis, MO). Plasma concentrations were measured by comparison of peak areas of individual fatty acids with the peak area of the palmitoyl-D31-fatty acid methyl ester internal standard using individual calibration curves for each of the 22 fatty acid methyl esters measured. The chromatographic peak for palmitoyl-D31-fatty acid methyl ester elutes about 1 minute earlier than non-labelled palmitoyl fatty acid methyl ester in a zone free of interference from other peaks. Each chromatogram was integrated automatically and checked for accuracy and specificity by a laboratory technician. Results were reported as mol% for each individual FA, taking into account the large difference in molecular mass between the shortest (myristic acid, 14:0) and longest (docosapentaenoic acid, 22:5n-3) chain FA measured. Analytical quality control was carried out by the daily use of standard quality control plasma samples. The CVs for the major fatty acids were between 3% to 13%. |
| **Outcome Assessment** | | Mortality Definitions  And Maximum Follow-up Time for the four major mortality outcomes | All participants are flagged for death certification with the National Health Service Central Register, UK with death certificates coded by nosologists according to the International Classification of Disease (ICD). Deaths due to cardiovascular diseases, cancer, and other causes were defined using ICD9 or ICD10 codes as underlying cause of death. Deaths due to cardiovascular diseases were defined as a death due to either coronary heart disease or stroke. The mortality data up to 31 December 2013 were collected. |
|  | |  |  |
|  | |  | **FHS** |
| **Cohort** | | Full Name | Framingham Heart study - Offspring cohort |
| **Descriptive + Acknowledgement** | | Field centers/Cities, State; Country (Sample source) | Framingham, MA, USA |
|  |  | Description | Our analysis focused on the Framingham Heart Study (FHS) Offspring sample, a population based longitudinal study of families living in Framingham, Massachusetts. The offspring study was initiated in 1971 and consisted of a sample of 5,124 individuals, offspring of the original cohort and their spouses. |
|  |  | References | Kannel WB, Feinleib M, McNamara PM, Garrison RJ, Castelli WP. An investigation of coronary heart disease in families. The Framingham offspring study. Am J Epidemiol. 1979;110:281–290. |
|  |  | Contact | William Harris (wsh@faresinst.com) |
|  |  | Acknowledgements | The authors express their gratitude to the FHS participants. |
|  |  | Funding Information | The Framingham Heart Study is conducted and supported by the National Heart, Lung and Blood Institute (NHLBI) and in collaboration with Boston University (Contract No. N01-HC-25195). |
| **Ascertainment of Fatty Acid Biomarker Concentrations** | | Please provide a brief description of methods used to ascertain fatty acid concentrations, including laboratory CVs | The fatty acid composition of RBC samples were analyzed by gas chromatography equipped with a SP 2560 capillary column after direct transesterification for 10 minutes in boron trifluoride/ methanol and hexane at 100 C as previously described. This technique generates fatty acids primarily from RBC glycerophospholipids. RBCs were isolated from blood drawn after a 10–12 h fast and frozen at −80 °C immediately after collection. All fatty acids present at >1% abundance had CVs of ≤7%. |
| **Outcome Assessment** | | Mortality Definitions  And Maximum Follow-up Time for the four major mortality outcomes | Mortality is ascertained annually, with data through 2014 available for this analysis. All identified events are adjudicated by the FHS Endpoint review committee and represent a combination of both active and passive ascertainment. In particular, information about CVD events on follow-up was obtained with the aid of medical histories, physical examinations at the study clinic, hospitalization records, and communication with personal physicians (D’Agostino, et al. 2008). |

|  | |  | **Hisayama** | |
| --- | --- | --- | --- | --- |
| **Cohort** | | Full Name | The Hisayama Study | |
| **Descriptive + Acknowledgement** | | Field centers/Cities, State; Country (Sample source) | Hisayama town, Kasuya-gun, Fukuoka prefecture, Japan | |
|  |  | Description | The Hisayama Study is an ongoing, population-based prospective cohort study of cardiovascular disease and its risk factors in the town of Hisayama, a suburb in the metropolitan in Japan. A total of 3293 residents who were aged 40 years or older and had no missing values for serum fatty acid levels were enrolled in the present study. | |
|  |  | References | [1. Ninomiya T, Nagata M, Hata J, Hirakawa Y, Ozawa M, Yoshida D, Ohara T, Kishimoto H, Mukai N, Fukuhara M, Kitazono T, Kiyohara Y. Association between ratio of serum eicosapentaenoic acid to arachidonic acid and risk of cardiovascular disease: the Hisayama Study. Atherosclerosis. 2013 Dec;231(2):261-7.](http://www.chs-nhlbi.org/%20Ann%20Epidemiol.%201(3):%20263-276,%201991%20Fried,%20L.P.%20et%20al.%20The%20Cardiovascular%20Health%20Study:%20design%20and%20rationale.%20Ann%20Epidemiol%201,%20263-76%20(1991).) | |
|  |  | Contact | Toshi Ninomiya (nino@eph.med.kyushu-u.ac.jp) | |
|  |  | Acknowledgements | The authors thank the staff of the Division of Health and Welfare of Hisayama for their cooperation in this study. | |
|  |  | Funding Information | This study was supported in part by Grants-in-Aid for Scientific Research (A) (JP16H02692) and (B) (JP16H05850, JP17H04126, and JP18H02737) and (C) (JP17K09114, JP17K09113, JP17K01853, JP18K07565, JP18K09412,and JP19K07890) and (Early-Career Scientists) (JP18K17925, and JP18K17382) from the Ministry of Education, Culture, Sports, Science and Technology of Japan; by Health and Labour Sciences Research Grants of the Ministry of Health, Labour and Welfare of Japan (H29-Junkankitou-Ippan-003, and H30-Shokuhin-[Sitei]-005); and by the Japan Agency for Medical Research and Development (JP19dk0207025, JP19ek0210082, JP19ek0210083, JP19km0405202, JP19ek0210080, JP19fk0108075). | |
| **Ascertainment of Fatty Acid Biomarker Concentrations** | | Please provide a brief description of methods used to ascertain fatty acid concentrations, including laboratory CVs | Serum fatty acids levels were assayed by gas chromatography (SRL, Tokyo, Japan). Briefly, total lipids in plasma were extracted according to the Folch’s procedure, followed by hydrolysis to free fatty acids. Free fatty acids were esterified with potassium methoxide/methanol and boron trifluoride methanol. The methylated fatty acids were analyzed using GC-17A gas chromatograph (Shimadzu Corporation, Kyoto, Japan) with omegawax-250 capillary column (SUPELCO, Sigma Aldrich Japan, Tokyo, Japan). Reproducibility (i.e. the coefficient of variation) of the determination of serum EPA, DHA, and AA levels by this method was reported to be 4.4%, 2.3%, and 3.8%, respectively.  Ozawa A, Takayanagi K, Fujita T, et al. Determination of long chain fatty acids in human total plasma lipids using gas chromatography [in Japanese]. Jpn Analyst 1982;31:87e91 cited by, https://www.jstage.jst.go.jp/article/bunsekikagaku1952/31/2/31_2_87/_article/-char/en/. | |
| **Outcome Assessment** | | Mortality Definitions  And Maximum Follow-up Time for the four major mortality outcomes | The subjects were followed up prospectively for 10 years from 2002 to November 2012 by repeated health examinations and by a daily monitoring system established by the study team and local physicians or members of the town’s Health and Welfare Office. Vital status was checked once yearly by mail or telephone for any subjects who did not undergo a regular examination or who moved out of town. Information about death was received from this follow-up system. When the subject died, we collected all medical information related to his/her illness and death, including hospital charts, physician’s records, and death certificate. Moreover, an autopsy was performed at the Department of Pathology of Kyushu University, if consent to autopsy was obtainable. All participants were followed up completely over 10 years. All the medical data including autopsy findings were scrutinized, and the underlying causes of death were classified according to the International Classification of Diseases, 10th revision (ICD-10). The outcome considered in this analysis was cardiovascular death (ICD-10: I00-I99), deaths from cancer (ICD-10: C00-C99), deaths from respiratory diseases (ICD-10: J00-J99.8), and deaths from other causes. | |
|  |  | | | **HPFS** |
| **Cohort** | Full Name | | | Health Professionals Follow-up Study (HPFS) |
| **Descriptive + Acknowledgement** | Field centers/Cities, State; Country (Sample source) | | | United States |
|  | Description | | | The Health Professionals Follow-up Study (HPFS) started in 1986, with 51,529 male health professionals, who were 40 – 75 years of age at recruitment in 1986. Blood samples were collected from HPFS participants in 1994. For this study we utilized previously measured fatty acid concentrations in stored blood used for nested case-control studies of incident cardiovascular diseases. Subjects were free of cardiovascular diseases, cancer and diabetes at the time of blood sampling. A complete-case analysis was performed via sampling of the case-control subsets and after excluding participants without covariate information. |
|  | References | | | Rimm EB, Giovannucci E, Stampfer MJ, Colditz GA, Litin LB, Willett WC. Reproducibility and validity of an expanded self-administered semiquantitative food frequency questionnaire among male health professionals. Am J Epidemiol.1992;135:1114-1126. |
|  | Contact | | | Qi Sun (qisun@hsph.harvard.edu) |
|  | Acknowledgements | | | The authors would like to thank the participants and staff of the HPFS for their dedication and contribution to the research and the following state cancer registries for their help: AL, AZ, AR, CA, CO, CT, DE, FL, GA, ID, IL, IN, IA, KY, LA, ME, MD, MA, MI, NE, NH, NJ, NY, NC, ND, OH, OK, OR, PA, RI, SC, TN, TX, VA, WA, WY. |
|  | Funding Information | | | This work was supported by grants from the National Institutes of Health (U01 CA167552, R01 HL35464, and T32 CA009001 [to Andres V. Ardisson Korat). |
| **Ascertainment of Fatty Acid Biomarker Concentrations** | Please provide a brief description of methods used to ascertain fatty acid concentrations, including laboratory CVs | | | Blood samples were sent to the lab with an ice pack via overnight courier and the majority of the samples arrived within 24 hours. Fifty nine percent of the study participants provided fasting blood samples. Upon arrival, samples were centrifuged and divided into aliquots for plasma, white blood cell, and red blood cells, and stored in liquid nitrogen freezers at ≤-130°C. Fatty acid concentrations were measured in stored total plasma and erythrocyte samples using gas-liquid chromatography. Concentrations of individual circulating fatty acids were expressed as a percentage of total fatty acids either in plasma or erythrocyte membranes The average intra-assay CV were 10% for erythrocyte linoleic acid, 10% for erythrocyte arachidonic acid, 7% for plasma linoleic acid, and 10% for plasma arachidonic acid. |
| **Outcome Assessment** | Mortality Definitions  And Maximum Follow-up Time for the four major mortality outcomes | | | Notification of the death of a cohort member may be received from next-of-kin or from the post office when a questionnaire or newsletter mailed to a participant is returned. Deaths are also identified through searches of the National Death Index. Once a death is reported, attempts are made to contact the next-of-kin or other contact person to learn the cause of death and to get permission to obtain medical records. Information may also be sought from the National Death Index, from tumor registries, and from death certificates. All information is reviewed by cohort investigators who determine the primary cause of death. Maximum follow-up time is 25 years. |

|  |  | **KIHD** |
| --- | --- | --- |
| **Cohort** | Full Name | Kuopio Ischaemic Heart Disease Risk Factor Study (KIHD) |
| **Descriptive + Acknowledgement** | Field centers/Cities, State; Country (Sample source) | Eastern Finland |
|  | Description | The KIHD study was designed to investigate risk factors for CVD, atherosclerosis, and related outcomes in a population-based, randomly selected sample of men from eastern Finland. The baseline examinations were carried out in 1984-1989. A total of 2682 men who were 42, 48, 54 or 60 years old at baseline (82.9% of those eligible) were recruited in two cohorts. The first cohort consisted of 1166 men who were 54 years old, enrolled in 1984-1986, and the second cohort included 1516 men who were 42, 48, 54 or 60 years old, enrolled in 1986-1989. The baseline examinations were followed by the 4-year examination round (1991-1993) in which 1038 men from the second cohort (88% of the eligible) participated. At the 11-year examination round (1998-2001), all men from the second cohort were invited and 854 men (95% of the eligible) participated. These examinations were also the baseline for 920 postmenopausal women (78.4% of the 1173 eligible women) from the same area, aged 53-73 years. 1123 eligible participants with data on serum fatty acids from the 11-y examinations were included in the current analyses. |
|  | References | Salonen JT. Is there a continuing need for longitudinal epidemiologic research? The Kuopio Ischaemic Heart Disease Risk Factor Study. Ann Clin Res. 1988; 20: 46-50. |
|  | Contact | Jyrki Virtanen (jyrki.virtanen@uef.fi) |
|  | Funding Information | The Kuopio Ischaemic Heart Disease Risk Factor Study (KIHD) was supported mainly by the funding from the Academy of Finland to Jukka T. Salonen. |
| **Ascertainment of Fatty Acid Biomarker Concentrations** | Please provide a brief description of methods used to ascertain fatty acid concentrations, including laboratory CVs | Venous blood samples were collected between 8AM and 10AM after an overnight fast. Serum total fatty acids were determined from frozen samples with a NB-351 capillary column (HNU-Nordion, Helsinki, Finland) by a Hewlett-Packard 5890 Series II gas chromatograph (Hewlett-Packard Company, Avondale, Pa, USA, since 1999 Agilent Technologies Inc., USA) with a flame ionization detector. Serum was extracted with chloroform-methanol and fatty acids were methylated with methanol and sulphuric acid prior to gas chromatography. Each analyte had an individual reference standard and the analytes were quantified with an internal standard method using eicosane. The coefficient of variation (CV%) for repeated measurements of fatty acids was 5.8% for alpha-linolenic acid (ALA, 18:3n-3), 5.9% for eicosapentaenoic acid (EPA, 20:5n-3), 9.2% for docosapentaenoic acid (DPA, 22:5n-3), and 5.7% for docosahexaenoic acid (DHA, 22:6n-3). |
| **Outcome Assessment** | Mortality Definitions  And Maximum Follow-up Time for the four major mortality outcomes | Deaths were ascertained by a computer linkage to the national cause of death register using the Finnish personal identification code (social security number). All deaths were coded according to the Tenth International Classification of Disease (ICD) codes. |

|  |  | **MCCS** |
| --- | --- | --- |
| **Cohort** | Full Name | Melbourne Collaborative Cohort Study (MCCS) |
| **Descriptive + Acknowledgement** | Field centers/Cities, State; Country (Sample source) | Melbourne, Victoria, Australia |
|  | Description | The Melbourne Collaborative Cohort Study (MCCS) is a prospective cohort study of 41,513 women and men aged 27 to 75 years (99% were between 40-69 years) when recruited between 1990 and 1994 [Milne et al. Int J Epidemiol, 2017]. Italian and Greek migrants were over-sampled to extend the range of lifestyle exposures. Participants were recruited via the electoral rolls (registration to vote is compulsory for adults in Australia), advertisements, and community announcements in local media (e.g. television, radio, and newspapers). Comprehensive lists of Italian and Greek surnames in the phone book and Electoral Rolls were also used to target southern European migrants. The Cancer Council Victoria’s Human Research Ethics Committee approved the study protocol. Participants gave written consent to participate and for the investigators to obtain access to their medical records. Vital status and cause of death information were obtained via linkage to the National Death Index of Australia. We used a case-cohort design to measure plasma fatty acids using baseline blood samples. For this analysis we have only included all members who were randomly selected (from the whole of the MCCS baseline sample) for the subcohort (N=4659). After exclusions we were left with 3,796 participants for the all-cause mortality analyses and 3,778 participants for the cause-specific mortality analyses. |
|  | References | [Milne RL, Fletcher AS, MacInnis RJ, Hodge AM, Hopkins AH, Bassett JK, Bruinsma FJ, Lynch BM, Dugué PA, Jayasekara H, Brinkman MT, Popowski LV, Baglietto L, Severi G, O'Dea K, Hopper JL, Southey MC, English DR, Giles GG. Cohort Profile: The Melbourne Collaborative Cohort Study (Health 2020). Int J Epidemiol. 2017 Dec 1;46(6):1757-1757i. doi: 10.1093/ije/dyx085. PubMed PMID: 28641380.](http://www.chs-nhlbi.org/%20Ann%20Epidemiol.%201(3):%20263-276,%201991%20Fried,%20L.P.%20et%20al.%20The%20Cardiovascular%20Health%20Study:%20design%20and%20rationale.%20Ann%20Epidemiol%201,%20263-76%20(1991).) |
|  | Contact | Allison Hodge (Allison.Hodge@cancervic.org.au) |
|  | Acknowledgements | The MCCS was made possible by the contribution of many people, including the original investigators, the teams that recruited the participants and continue working on follow-up, and the many thousands of Melbourne residents who continue to participate in the study. |
|  | Funding Information | Cohort recruitment was funded by Cancer Council Victoria [http://www.cancervic.org.au/] and VicHealth [https://www.vichealth.vic. gov.au/]. The MCCS was further supported by grants 209057, 251553 and 504711 from the Australian National Health and Medical Research Council (NHMRC) [http://www.nhmrc.gov.au/] and ongoing follow-up and data management has been funded by Cancer Council Victoria since 1995. |
| **Ascertainment of Fatty Acid Biomarker Concentrations** | Please provide a brief description of methods used to ascertain fatty acid concentrations, including laboratory CVs | Plasma phospholipid fatty acids were measured at the Fred Hutchinson Cancer Research Center (Seattle, WA) using stored blood samples from 1992-1993. Total lipids were extracted from plasma using the methods of Folch. A one dimensional thin-layer chromatography was used to separate phospholipids from neutral lipids. Phospholipids fraction was directly trans-esterified using the Lepage and Roy method to prepare fatty acid methyl esters, and individual fatty acid methyl esters were separated using gas chromatography (Agilent 5890 Gas Chromatograph flame ionization detector, Agilent Technologies, Palo Alto, CA; fused silica capillary column SP-2560 [100m x 0.25mm, 0.2μm], Supelco Belefonte, PA; initial 160 degrees Celsius for 16 min, ramp 3 degrees Celsius/min to 240 degrees Celsius, hold 15 minutes). All fatty acids were processed at the Biomarker Laboratory of the Fred Hutchinson Cancer Research Center (Seattle, WA). For this analysis, levels of each individual fatty acid are expressed as a weight percentage of total phospholipid fatty acids analyzed. Inter-assay coefficient of variations for the VLSFA measurements were ≤ 3.5%. |
| Outcome Assessment | Mortality Definitions  And Maximum Follow-up Time for the four major mortality outcomes | Deaths were identified by at least annual linkage to the Victorian Registry of Births, Deaths and Marriages (considered complete) and by at least 2-yearly record linkage to the National Death Index (NDI), compiled by the Australian Institute of Health and Welfare (to which all state and territory death registries contribute). Linked NDI data also include cause of death. For the MCCS we had a longer follow-up for all-cause mortality: 902 deaths, N=3796 for analysis by end of FU (31Dec2016. For the cause-specific analysis: 646 deaths and N=3778; end of FU (31Dec2013). |

|  |  | **MESA** |
| --- | --- | --- |
| **Cohort** | Full Name | Multi-Ethnic Study of Atherosclerosis (MESA) |
| **Descriptive + Acknowledgement** | Field centers/Cities, State; Country (Sample source) | ^ U.S. communities: Wake Forest (NC), UCLA (CA), Northwestern University (IL), University of Minnesota (MN), Columbia (NY), Johns Hopkins (MD) |
|  | Description | The Multi-Ethnic Study of Atherosclerosis (MESA) is a study of the characteristics of subclinical cardiovascular disease (disease detected non-invasively before it has produced clinical signs and symptoms) and the risk factors that predict progression to clinically overt cardiovascular disease or progression of the subclinical disease. MESA researchers study a diverse, population-based sample of 6,814 asymptomatic individuals of European- African- Hispanic- and Chinese American ancestry ascertained across six field centers across the United States. Baseline data for the current analyses were taken from the first clinic exam conducted in 2000 – 2002. In addition to yearly phone calls, follow-up clinic exams are conducted approximately every two years, and at the time the current analyses were conducted incident diabetes was available until the fifth clinic exam conducted in 2010-2012. The sample included in this study was composed of 2,262 participants who had available fatty acid and follow-up diabetes information (2,234 free from diabetes at baseline) |
|  | References | [Bild DE, Bluemke DA, Burke GL, Detrano R, Diez Roux AV, Folsom AR, et al. Multi-Ethnic Study of Atherosclerosis: Objectives and design. Am J Epidemiol. 2002;156(9):871–81. pmid:12397006 (1991).](http://www.chs-nhlbi.org/%20Ann%20Epidemiol.%201(3):%20263-276,%201991%20Fried,%20L.P.%20et%20al.%20The%20Cardiovascular%20Health%20Study:%20design%20and%20rationale.%20Ann%20Epidemiol%201,%20263-76%20(1991).) |
|  | Contact | Mike Tsai (tsaix001@tc.umn.edu) |
|  | Acknowledgements | The authors thank the other investigators, the staff, and the participants of the MESA study for their valuable contributions. A full list of participating MESA investigators and institutions can be found at http://www.mesa-nhlbi.org. |
|  | Funding Information | MESA was supported by contracts HHSN268201500003I, N01-HC-95159, N01-HC-95160, N01-HC-95161, N01-HC-95162, N01-HC-95163, N01-HC-95164, N01-HC-95165, N01-HC-95166, N01-HC-95167, N01-HC-95168 and N01-HC-95169 from the National Heart, Lung, and Blood Institute, and by grants UL1-TR-000040, UL1-TR-001079, and UL1-TR-001420 from NCATS. |
| **Ascertainment of Fatty Acid Biomarker Concentrations** | Please provide a brief description of methods used to ascertain fatty acid concentrations, including laboratory CVs | Fatty acids were measured in EDTA plasma frozen at -70˚C using samples collected after a 12-hour fast [33, 34]. Plasma phospholipids were isolated by thin layer chromatography, with FAs being subsequently separated by gas chromatography. The Collaborative Studies Clinical Laboratory at Fairview-University Medical Center (Minneapolis, MN) performed the FA assays. Individual FAs were expressed as a percentage of total FAs. |
| **Outcome Assessment** | Mortality Definitions  And Maximum Follow-up Time for the four major mortality outcomes | Participants are followed up for incident cardiovascular events, including a diagnosis of CVD, at yearly intervals from the baseline exam. In addition to follow-up MESA study visits, a telephone interviewer contacts each participant yearly to inquire about all interim hospital admissions, cardiovascular outpatient diagnoses, and deaths. Copies of all death certificates and medical records for all hospitalizations and outpatient cardiovascular diagnoses are obtained; alongside next-of-kin interviews were appropriate. Over 95% of hospital records are obtained, with trained personnel abstracting any medical records suggesting possible cardiovascular events. Two physicians independently review all medical records for end point classification and assignment of incidence dates. In the rare case of disagreements, differences are adjudicated and moved to a full committee if resolution is not obtained. |

|  |  | | **METSIM** | |
| --- | --- | --- | --- | --- |
| **Cohort** | Full Name | | METabolin SYndrome In Men (METSIM) | |
| **Descriptive + Acknowledgement** | | Field centers/Cities, State; Country (Sample source) | Finland, Kuopio |  |
|  |  | Description | 10,197 men, aged from 45 to 73 years at baseline, about 6,700 of them have follow-up data |  |
|  |  | References | Laakso M, Kuusisto J, Stančáková A, Kuulasmaa T, Pajukanta P, Lusis AJ, Collins FS, Mohlke KL, Boehnke M. [The Metabolic Syndrome in Men study: a resource for studies of metabolic and cardiovascular diseases.](https://linkprotect.cudasvc.com/url?a=https%3a%2f%2fpubmed.ncbi.nlm.nih.gov%2f28119442%2f%3ffrom_term%3dlaakso%2bm%2band%2bjournal%2bof%2blipid%2bresearch%26from_sort%3ddate%26from_pos%3d2&c=E,1,eepAqAaItHSj_UZ0T0rKT9ulr1XCTSAOUBelu_rbWf4M2PeXkBqtKhTEcCcDu4pvbR9kTNhfZRRHRHnyFNC7zR50zA_iHG3KtmOiedOA50nKAAaz2w,,&typo=1) J Lipid Res. 2017. 58:481-493. |  |
|  |  | Contact | Markko Laasko (Markku.laakso@uef.fi) |  |
|  |  | Funding Information | METSIM has been funded by grants from the Academy of Finland, European Commission, National Institute of Health, Sigrid Juselius Foundation, Finnish Foundation for Cardiovascular Research, and Kuopio University Hospital. |  |
| **Ascertainment of Fatty Acid Biomarker Concentrations** | Please provide a brief description of methods used to ascertain fatty acid concentrations, including laboratory CVs | | Lipids were extracted from plasma sample with chloroform-methanol (2:1) and lipid fractions were separated with an aminopropyl column. FAs in lipid fractions were transmethylated with 14% borontrifluoride in methanol. Finally, FA methyl esters were analyzed by 7890A gas-chromatograph (Agilent Technologies, Inc., Wilmington, DE, USA) equipped with a 25-m free FA phase column (Agilent Technologies). Phosphatidylcholine dinonadecanoyl (Larodan Fine Chemicals, Malmo, Sweden) served as an internal standard. | |
| **Outcome Assessment** | Mortality Definitions and Maximum Follow-up Time for the four major mortality outcomes | | From different registries: mortality, cardiovascular events, new cases of type 2 diabetes, etc. Baseline study done in 2005-2010, and follow-up studies starting in 2010, and still continuing. | |

|  |  | **NHS** |
| --- | --- | --- |
| **Cohort** | Full Name | Nurses’ Health Study (NHS) |
| **Descriptive + Acknowledgement** | Field centers/Cities, State; Country (Sample source) | United States |
|  | Description | NHS was established in 1976 by recruiting 121,700 female nurses aged 30 to 55 who responded to a questionnaire with information related to their health, lifestyle practices and occurrence of chronic diseases. Blood samples were collected from NHS participants in 1989-1990. For this study we utilized previously measured fatty acid concentrations in stored blood used for nested case-control studies of incident cardiovascular diseases. Subjects were free of cardiovascular diseases, cancer and diabetes at the time of blood sampling. A complete-case analysis was performed via sampling of the case-control subsets and after excluding participants without covariate information. |
|  | References | 1. Belanger CF, Hennekens CH, Rosner B, Speizer FE. The Nurses' Health Study. Am J Nurs 1978;78(6):1039-40. PubMed PMID: 248266 2. Bao Y, Bertoia ML, Lenart EB, Stampfer MJ, Willett WC, Speizer FE, Chavarro JE. Origin, Methods, and Evolution of the Three Nurses' Health Studies. Am J Public Health. 2016 Sep;106(9):1573-81. |
|  | Contact | Frank Hu (fhu@hsph.harvard.edu) |
|  | Acknowledgements | The authors would like to thank the participants and staff of the NHS for their dedication and contribution to the research and the following state cancer registries for their help: AL, AZ, AR, CA, CO, CT, DE, FL, GA, ID, IL, IN, IA, KY, LA, ME, MD, MA, MI, NE, NH, NJ, NY, NC, ND, OH, OK, OR, PA, RI, SC, TN, TX, VA, WA, WY. |
|  | Funding Information | This work was supported by grants from the National Institutes of Health (UM1 CA186107, P01 CA87969, R01 CA49449, R01 HL034594, and T32 CA009001 [to Andres V. Ardisson Korat). |
| **Ascertainment of Fatty Acid Biomarker Concentrations** | Please provide a brief description of methods used to ascertain fatty acid concentrations, including laboratory CVs | Blood samples were sent to the lab with an ice pack via overnight courier and the majority of the samples arrived within 24 hours. Seventy one percent of the study participants provided fasting blood samples. Upon arrival, samples were centrifuged and divided into aliquots for plasma, white blood cell, and red blood cells, and stored in liquid nitrogen freezers at ≤-130°C. Fatty acid concentrations were measured in stored total plasma and erythrocyte samples using gas-liquid chromatography. Concentrations of individual circulating fatty acids were expressed as a percentage of total fatty acids either in plasma or erythrocyte membranes. CVs were 10% for linoleic acid and arachidonic acid for erythrocyte membrane, 7% for linoleic in plasma, and 10% for arachidonic acid in plasma. |
| **Outcome Assessment** | Mortality Definitions  And Maximum Follow-up Time for the four major mortality outcomes | Notification of the death of a cohort member may be received from next-of-kin or from the post office when a questionnaire or newsletter mailed to a participant is returned. Deaths are also identified through searches of the National Death Index. Once a death is reported, attempts are made to contact the next-of-kin or other contact person to learn the cause of death and to get permission to obtain medical records. Information may also be sought from the National Death Index, from tumor registries, and from death certificates. All information is reviewed by cohort investigators who determine the primary cause of death. Maximum follow-up time is 29 years. |

|  |  | **Three-City** |
| --- | --- | --- |
| **Cohort** | Full Name | Three-City (3C) study |
| **Descriptive + Acknowledgement** | Field centers/Cities, State; Country (Sample source) | 3 French cities: Bordeaux, Dijon, Montpellier |
|  | Description | The Three-City (3C) study is an ongoing multicenter prospective cohort study of vascular risk factors for dementia which started in 1999-2000 and included 9,294 community dwellers in three French cities: Bordeaux (n=2,104), Dijon (n=4,931) and Montpellier (n=2,259). Individuals living in one of these cities, aged 65 years and over and not institutionalized were eligible for recruitment into the 3C study. The protocol of the 3C study has been approved by the Consultative Committee for the Protection of Persons participating in Biomedical Research of the Kremlin-Bicêtre University Hospital (Paris). All participants gave their written informed consent. The baseline data collection included socio-demographic and lifestyle characteristics, symptoms and complaints, main chronic conditions, medication use, neuropsychological testing, clinical examination including blood pressure measurement, electrocardiogram (ECG) and blood sampling. Total plasma fatty acids were measured at baseline from fasting blood samples among 1,416 individuals from the Bordeaux centre |
|  | References | <https://www.ncbi.nlm.nih.gov/pubmed/14598854> |
|  | Contact | Cecelia Samieri (cecilia.samieri@u-bordeaux.fr) |
|  | Funding Information | The Three-City Study is conducted under a partnership agreement between Sanofi-Aventis, the Institut National de la Santé et de la Recherche Médicale (INSERM) and the Institut de Santé Publique et Développement of the Victor Segalen Bordeaux 2 University. The Fondation pour la Recherche Médicale (FRM) funded the preparation and initiation of the study. The 3C Study is also supported by the Caisse Nationale Maladie des Travailleurs Salariés (CNAMTS), Direction Générale de la Santé (DGS), Mutuelle Générale de l’Education Nationale (MGEN), Institut de la Longévité, Regional Councils of Aquitaine and Bourgogne, Fondation de France, and Ministry of Research - INSERM Programme “Cohortes et collections de données biologiques.” The 10-year follow-up has been financed by ANR 2007LVIE 003 and the “Fondation Plan Alzheimer”. |
| **Ascertainment of Fatty Acid Biomarker Concentrations** | Please provide a brief description of methods used to ascertain fatty acid concentrations, including laboratory CVs | In the 3C study, fasting blood samples were collected at the baseline visit into heparinized evacuated tubes and centrifuged at 1000 _ g for 10 min. Total lipids were extracted from plasma with 5 mL of hexane/isopropanol (3:2, by vol). The plasma fatty acid composition was determined from 2 mL of the lipid extract after transformation into isopropyl esters. Separation of isopropyl esters was made on a gas chromatograph (Trace, Thermoelectron, France) using a 25-m Carbowax capillary column (internal diameter: 0.32 mm). Column conditions were 180 °C for 5 min, increasing by 7.5 °C/min to 220 °C for 30 min. The injector was set at 60 °C and the flame ionization detector at 250 °C. Helium was used as the carrier gas (flow rate: 2 mL/min). The peaks were identified by comparison with reference fatty acid esters (Sigma, St Louis, MO), and peak areas were measured with an automatic integrator (DP700; Fisons Instruments, France). The results for each fatty acid were expressed as a percentage of total fatty acids. |
| **Outcome Assessment** | Mortality Definitions  And Maximum Follow-up Time for the four major mortality outcomes | After the baseline examination, participants were assessed 7 times every 2 to 3 years. The vital status of each participant over the follow-up was determined based on death certificates, medical records and reports from the participant’s family and physician. A committee reviewed records to determine immediate and underlying causes of death, according to the International Classification of Diseases (ICD) codes, 10th revision. The vital status was confirmed over the 17-year of follow-up, and underlying cases of deaths were determined until 10-year follow-up. Our analyses Death from CVD was defined as an immediate cause of death with ICD codes between I00 and I99 (including coronary heart disease, stroke, peripheral vascular disease and other cardiovascular disease), or equal to R09.2 or R96.0. Death from cancer as an immediate cause of death included ICD codes between C00 and C97. Other causes of deaths included infectious and respiratory diseases, digestive causes, trauma, suicide and death of undetermined cause. |

| **Cohort** | Full Name | | **Uppsala Longitudinal Study of Adult Men (ULSAM)** | |
| --- | --- | --- | --- | --- |
| **Descriptive + Acknowledgement** | Field centers/Cities, State; Country (Sample source) | | Uppsala county, Sweden | |
|  | Description | | ULSAM is a community-based cohort of men living in Uppsala county, Sweden. The origin of this longitudinal study was the "Uppsala Primary Preventive Study", carried out between September 1970 and September 1973. The study comprised all men living in the County of Uppsala born between 1920 and 1924 selected from the register of County Council. All men (n=2841) were invited for the investigation, 81.7% (n=2322) participated. The mean age at this baseline examination was 49.6 (SD +/- 0.6), hence this starting cohort was referred to as ULSAM-50. After this baseline examination, all men were invited to participate in follow-up investigations at the ages 70, 82 and 88. Between the age 50 and 70, 422 had died and 219 had moved out of the Uppsala region. Of the 1681 men invited, 460 did not participate in this follow up, leaving 1221 men who participated (response rate of 73%) aged around 70. The men were invited by a letter, which also explained the aim of the examination. They received the letter 7-10 days prior to the examination. Those born at the beginning of the year were called first. Six individuals were called every weekday except for the vacation period in Sweden between June 25 and August 15. A second invitation letter was sent at the end of the examination of each age class to those who had not come after the first invitation. The screening examination program included a medical questionnaire and interview, blood and urine sampling, blood pressure and anthropometric measurements, intravenous glucose tolerance test, ECG recording, chest X-ray and pure tone audiometry. At the baseline exam, fatty acid composition was assessed in serum cholesterol, whereas at the second exam 20 years later, fatty acids were measured in both cholesterol esters and adipose tissue. Dodecapenta and dodecahepta acids were measured only in adipose tissue lipids. | |
|  | References | | [Hagström E, Kilander L, Nylander R, Larsson E-M, Michaëlsson K, Melhus H, et al. Plasma parathyroid hormone is associated with vascular dementia and cerebral hyperintensities in two community-based cohorts. J Clin Endocrinol Metab. 2014;99(11):4181–9. doi:10.1210/jc.2014-1736 Huang X, Sjögren P, Ärnlöv J, Cederholm T, Lind L, Stenvinkel P, et al. Serum fatty acid patterns, insulin sensitivity and the metabolic syndrome in individuals with chronic kidney disease. Journal of internal medicine. 2014;275(1):71–83. doi:10.1111/joim.12130. Hedstrand H. A study of middle-aged men with particular reference to risk factors for cardiovascular disease. Upsala J Med Sci. 1975;19:1–61.](http://www.chs-nhlbi.org/%20Ann%20Epidemiol.%201(3):%20263-276,%201991%20Fried,%20L.P.%20et%20al.%20The%20Cardiovascular%20Health%20Study:%20design%20and%20rationale.%20Ann%20Epidemiol%201,%20263-76%20(1991).) | |
|  | Contact | | Ulf Riserius (ulf.riserus@pubcare.uu.se) | |
|  | Funding Information | | The Uppsala Longitudinal Studies of Adult Men 50 and 70 were funded by Uppsala City Council; The Swedish Research Council (K2015-54X-22081-04-3); and The Swedish Research Council for Environment, Agricultural Sciences and Spatial Planning (2016-01639). Dr Marklund is a researcher within a National Health and Medical Research Council Centre for Research Excellence in reducing salt intake using food policy interventions (APP1117300) | |
| **Ascertainment of Fatty Acid Biomarker Concentrations** | Please provide a brief description of methods used to ascertain fatty acid concentrations, including laboratory CVs | | For analysis of the fatty acid composition of the serum cholesterol esters in ULSAM-50, serum was extracted with a hexane-isopropanol solution (1+4).58 Cholesterol esters were separated from the extract by thin layer chromatography before inter-esterification (acidic methanol at 85°C, 2 h),59 and free cholesterol liberated in the reaction was removed by an aluminum oxide column to avoid contamination of the gas liquid chromatography column. The percentage composition of methylated fatty acids 14:0 to 22:6 was determined by gas chromatography (a 25 m NB-351 silica capillary column, i.d. 0.32 mm, phase layer 0.20 mm) with use of a flame ionisation detector and with helium as carrier gas. Every 25th sample was a serum control pool. The precision of the between-series analysis (n=35) varied from 2% (large peaks) to 10% (smaller peaks) and between successive gas chromatography runs (n=17). Intraassay CV: 0.2-5% depending on the fatty acid; Interassay: 2-10% depending on the fatty acid. For concentrations below detection limit, proportions were imputed as a random value between 0 and the minimum quantified proportion in the cohort. | |
| **Outcome Assessment** | Mortality Definitions  And Maximum Follow-up Time for the four major mortality outcomes | | Total and cause-specific mortality during follow-up was identified using the Swedish Cause of Death register. Using registry data, mortality was identified according to the International Classification of Disease 8th (ICD-8), 9th (ICD-9) and 10th revision (ICD-10). Deaths attributed to ICD-10 codes I00-I09, I11, I13, I20-I51, or I60-I70 (or corresponding ICD-8/9 codes) were defined as CVD deaths; ICD-10 codes C00-C97 (or corresponding ICD-8/9 codes) as cancer deaths, and all other causes of deaths categorized as other deaths. | |
|  | |  | | **WHIMS** |
| **Cohort** | | Full Name | | Women's Health Initiative Memory Study |
| **Descriptive + Acknowledgement** | | Field centers/Cities, State; Country (Sample source) | | 50 participating centers |
|  |  | Description | | WHIMS randomized trials which examined the effects of postmenopausal hormone therapy on cognitive function in women aged 65-80 years. Recruitment began in 1995. Global inclusion criteria included post-menopausal women between 50 and 79 years of age who were willing and able to provide written consent, and who planned to reside in the study recruitment for at least three years after enrollment. Global exclusion criteria included medical conditions that would be predictive of a survival of less than three years, possessing characteristics or conditions that may diminish study adherence (e.g., sub abuse, mental illness or cog impairment) or concurrent enrollment in another RCT. |
|  |  | References | | [PMID: 15213207, 9875839](http://www.chs-nhlbi.org/%20Ann%20Epidemiol.%201(3):%20263-276,%201991%20Fried,%20L.P.%20et%20al.%20The%20Cardiovascular%20Health%20Study:%20design%20and%20rationale.%20Ann%20Epidemiol%201,%20263-76(1991).) |
|  |  | Contact | | William Harris (wsh@faresinst.com) |
|  |  | Acknowledgements | | The WHI program is supported by contracts from the National Heart, Lung and Blood Institute, NIH. The authors thank the WHI investigators and staff for their dedication, and the study participants for making the program possible. A listing of WHI investigators can be found at http://www.whi.org/researchers/Documents%20%20Write%20a%20Paper/WHI%20Investigator %20Short%20List.pdf.” |
|  |  | Funding Information | | “The WHI program is funded by the National Heart, Lung, and Blood Institute, National Institutes of Health, U.S. Department of Health and Human Services through contracts HHSN268201600018C, HHSN268201600001C, HHSN268201600002C, HHSN268201600003C, and HHSN268201600004C |
| **Ascertainment of Fatty Acid Biomarker Concentrations** | | Please provide a brief description of methods used to ascertain fatty acid concentrations, including laboratory CVs | | The fatty acid composition of RBC samples were analyzed by gas chromatography equipped with a SP 2560 capillary column after direct transesterification for 10 minutes in boron trifluoride/ methanol and hexane at 100 C as previously described. This technique generates fatty acids primarily from RBC glycerophospholipids. During the aliquoting phase, the RBC samples were stored improperly at -20°C for a period of approximately 2 weeks, causing oxidative degeneration of the PUFAs before measurement. The original FA levels were estimated with multiple imputations using independent data on fatty acid degradation and length of time the samples were exposed to -20°C [Pottala et al. 2012]. All fatty acids present at >1% abundance had CVs of ≤6.5%. Pottala JV, Espeland MA, Polreis J, Robinson J, Harris WS (2012) “Correcting the effects of -20°C storage and aliquot size on erythrocyte fatty acid content in the Women’s Health Initiative” Lipids. 47(9):835-46. |
| **Outcome Assessment** | | Mortality Definitions  And Maximum Follow-up Time for the four major mortality outcomes | | Mortality and cause of mortality was determined through a central adjudication process as part of the WHIMS RCT. That process utilized data gathered from multiple sources such as autopsy/coroner’s report, medical records/ER records/EMS records, death certificate, informant interview, and the National Death Index. Median follow-up was 13 years for total and cause specific mortality. |

**Statistical Analysis**

*Covariates - Missing Data*

For missing covariate data, a missing indicator category were used for categorical covariates.  Missing continuous covariates were handled per the usual practice of each cohort and study investigators, e.g., imputation or exclusion.

*Meta analysis- Further details*

Primary reported meta-analyses were performed using fixed effects models, however we also conducted random-effects models (detailed results not shown) using the REML (restricted maximum-likelihood estimator; (1)) with no substantial differences in the pattern of results. All meta-analyses models, except the splines analysis, were conducted using the *metafor* package in R (2).

In the spline meta-analysis, we used risk estimates of study-specific quintile categories which were adjusted for covariates prior to meta-analysis (see footnote to Table 3 for full list). We determined the exposure dose to be the percentile scores calculated from medians of study-specific quintile categories. Using the continuous information of the percentile scores for each fatty acid of all participating cohorts, we obtained two spline terms, applying three knots of 10th, 50th and 90th percentiles of the dose distribution to a restricted cubic spline function. Then, using the spline terms as the exposure variables and risk estimates as the dependent variable, we conducted multivariable meta-analysis. The whole procedure was previously published by Orsini *et al*. (1) and implemented with Stata 15.1 (Stata Corp, College Station, TX) by modifying the script documented by Orsini et al. (4).

Then, summary estimates for spline terms were used to predict a non-linear dose-response curve with 95% confidence interval for which the reference value (risk ratio=1.0 with standard error=0) was specified to be 10^th^ percentile of the exposure distribution.

1. Viechtbauer, W., López-López, J. A., Sánchez-Meca, J., & Marín-Martínez, F. (2015). A comparison of procedures to test for moderators in mixed-effects meta-regression models. *Psychological Methods*, 20, 360–37.
2. Viechtbauer, W. (2010). Conducting meta-analyses in R with the metafor package. Journal of Statistical Software, 36(3), 1-48.
   <http://www.jstatsoft.org/v36/i03/>
3. Orsini N, Li R, Wolk A, Khudyakov P, Spiegelman D**.** Meta-Analysis for Linear and Nonlinear Dose-Response Relations: Examples, an Evaluation of Approximations, and Software. American Journal of Epidemiology. 2011;175(1):66-73
4. Orsini N, Bellocco R and Greenland S. Generalized Least Squares for Trend Estimation of Summarized Dose-response data. The Stata Journal. 6(1): 40-57.

**Table S2**. Means (SDs) of n-3 and n-6 fatty acids* by cohort and lipid fraction (percent of total fatty acids)

| Study† | Lipid fraction‡ | ALA | EPA | DPA | DHA | EPA+DHA | LA | AA |
| --- | --- | --- | --- | --- | --- | --- | --- | --- |
| 60YO | CE | 0.88 (0.20) | 2.07 (0.98) | NA | 0.91 (0.25) | 2.98 (1.17) | 48.47 (4.18) | 6.31 (1.17) |
| ULSAM50 | CE | 0.66 (0.16) | 1.35 (0.63) | NA | 0.70 (0.21) | 2.05 (0.77) | 53.99 (5.17) | 4.78 (0.94) |
| CCCC | Plasma | 0.67 (0.22) | 0.38 (0.25) | NA | 1.79 (0.65) | 2.17 (0.80) | 15.59 (4.38) | 3.05 (1.00) |
| Hisayama | Plasma | 0.69 (0.21) | 2.33 (1.30) | 0.65 (0.19) | 4.72 (1.32) | 7.05 (2.46) | 26.92 (4.55) | 5.00 (1.03) |
| KIHD | Plasma | 0.95 (0.28) | 1.63 (0.93) | 0.77 (0.16) | 2.72 (0.91) | 4.35 (1.73) | 24.55 (3.57) | 5.84 (1.20) |
| Three C | Plasma | 0.4 (0.17) | 1.05 (0.62) | 0.47 (0.17) | 2.47 (0.74) | 3.52 (1.17) | 25.47 (4.55) | 6.99 (1.62) |
| CSHA | RBC-PL | 0.04 (0.10) | 0.67 (0.40) | 2.05 (0.58) | 3.57 (1.24) | 4.25 (1.50) | 9.54 (1.88) | 12.90 (2.39) |
| FHS | RBC-PL | 0.18 (0.10) | 0.64 (0.32) | 2.65 (0.40) | 4.55 (1.25) | 5.19 (1.47) | 11.14 (1.64) | 16.7 (1.47) |
| HPFS | RBC-PL | 0.20 (0.21) | 0.49 (0.27) | 1.93 (0.40) | 3.56 (1.20) | 4.06 (1.41) | 13.00 (2.94) | 12.85 (1.85) |
| NHS | RBC-PL | 0.13 (0.06) | 0.41 (0.19) | 1.49 (0.33) | 2.93 (0.93) | 3.34 (1.05) | 12.03 (2.32) | 12.30 (1.95) |
| WHIMS | RBC-PL | 0.16 (0.07) | 0.70 (0.41) | 2.50 (0.43) | 4.50 (1.42) | 5.20 (1.67) | 11.82 (1.77) | 16.90 (1.75) |
| AGES-R | PL | 0.23 (0.08) | 2.88 (1.68) | 1.16 (0.21) | 6.33 (1.53) | 9.21 (3.02) | 17.5 (2.83) | 6.96 (1.59) |
| CHS | PL | 0.15 (0.05) | 0.59 (0.38) | 0.84 (0.17) | 3.05 (0.98) | 3.65 (1.22) | 19.72 (2.56) | 11.14 (1.94) |
| EPIC-Norfolk | PL | 0.23 (0.09) | 1.27 (0.83) | 1.40 (0.38) | 5.11 (1.62) | 6.38 (2.24) | 24.33 (3.42) | 9.47 (1.87) |
| MCCS | PL | 0.17 (0.08) | 1.05 (0.47) | 1.22 (0.24) | 3.98 (1.04) | 5.04 (1.30) | 20.20 (2.92) | 10.39 (1.76) |
| MESA | PL | 0.18 (0.07) | 0.99 (0.93) | 0.96 (0.23) | 4.20 (1.56) | 5.19 (2.26) | 21.50 (3.34) | 12.00 (2.58) |
| MetSIM | PL | 0.32 (0.12) | 2.29 (1.15) | 1.40 (0.21) | 5.66 (1.45) | 7.95 (2.36) | 18.61 (2.66) | 9.03 (1.62) |

*alpha-linolenic acid (ALA), eicosapentaenoic acid (EPA), docosapentaenoic acid (DPA), docosahexaenoic acid (DHA), linoleic acid (LA) and arachidonic acid (AA).

†60YO, Stockholm cohort of 60-year olds; AGESR: Age, Genes, Environment Susceptibility Study (Reykjavik); CCCC, Chin-Shan Community Cardiovascular Cohort Study; CHS, Cardiovascular Health Study; CSHA, Canadian Study of Health and Aging; EPIC-Norfolk, European Prospective Investigation into Cancer, Norfolk UK; FHS, Framingham Heart Study; HS, Hisayama Study; HPFS, Health Professionals Follow-up Study; KIHD, Kuopio Ischaemic Heart Disease; MCCS, Melbourne Collaborative Cohort Study; MESA, Multi-Ethnic Study of Atherosclerosis; MetSIM, Metabolic Syndrome in Men Study; NHS, Nurses Health Study; Three C, Three City Study; ULSAM, Uppsala Longitudinal Study of Adult Men; WHIMS, Women’s Health Initiative Memory Study. ‡ CE, cholesteryl esters; PL, phospholipids; RBC, red blood cell.

**Table S3**. **Deaths by cause and cohort**

| Cohort |  | Mortality | | | |
| --- | --- | --- | --- | --- | --- |
|  | Total N | Total^1^ | CVD | Cancer | Other |
| 60YO | 3659 | 756 | 197 | 328 | 231 |
| AGES-R | 1697 | 962 | 308 | 218 | 436 |
| CCCC | 1834 | 993 | 297 | 293 | 425 |
| CHS | 2256 | 1872 | 663 | 356 | 854 |
| CSHA | 424 | 19 | NA | NA | NA |
| EPIC-Norfolk | 6613 | 3347 | 864 | 1063 | 1420 |
| FHS | 2123 | 292 | 43 | 116 | 118 |
| Hisayama | 3293 | 469 | 122 | 145 | 109 |
| HPFS | 1477 | 878 | 304 | 166 | 408 |
| KIHD | 1125 | 310 | 121 | 86 | 103 |
| MCCS^2^ | 3796 | 902 | 138 | 303 | 205 |
| MESA | 1844 | 111 | 33 | NA | NA |
| MetSIM | 1354 | 58 | NA | 24 | NA |
| NHS | 1487 | 853 | 304 | 139 | 410 |
| 3-City^2^ | 1421 | 787 | 25 | 110 | 188 |
| ULSAM50 | 1878 | 1771 | 702 | 511 | 558 |
| WHIMS | 6185 | 1340 | 450 | 426 | 464 |
| Total | 39502 | 13989 | 3963 | 3979 | 5111 |

NA= death was not adjudicated for this cause
^1^ In some cases, total deaths are greater than the sum of CVD, cancer and other because some deaths were, at the time of database closure, of unknown cause. ^2^ For all cohorts except MCCS and 3-City study, cause-specific follow-up times were the same as for total mortality and are shown in Table 1. Median cause-specific mortality follow-up time for the 3-City Study was 10 years, and 20.3 years for MCCS.

**Table S4**. 10^th^ and 90^th^ percentiles of circulating n-3 fatty acids (% of total fatty acids in the lipid fraction) by cohort and by lipid fraction†

| Study | Lipid fraction | ALA | | EPA | | DPA | | DHA | | EPA+DHA | |
| --- | --- | --- | --- | --- | --- | --- | --- | --- | --- | --- | --- |
|  |  | **10^th^** | **90^th^** | **10^th^** | **90^th^** | **10^th^** | **90^th^** | **10^th^** | **90^th^** | **10^th^** | **90^th^** |
| 60YO | Cholesteryl Esters | 0.65 | 1.13 | 1.14 | 3.22 | NA | NA | 0.62 | 1.23 | 1.82 | 5.52 |
| ULSAM50 |  | 0.47 | 0.86 | 0.69 | 2.14 | NA | NA | 0.46 | 0.97 | 1.21 | 3.06 |
| CCCC | Plasma | 0.44 | 0.93 | 0.2 | 0.6 | NA | NA | 1.05 | 2.61 | 1.27 | 3.15 |
| Hisayama |  | 0.46 | 0.95 | 0.98 | 3.98 | 0.43 | 0.89 | 3.15 | 6.49 | 4.26 | 10.25 |
| KIHD |  | 0.63 | 1.32 | 0.77 | 2.71 | 0.57 | 0.98 | 1.67 | 3.93 | 2.57 | 6.51 |
| Three C |  | 0.2 | 0.6 | 0.5 | 1.8 | 0.3 | 0.6 | 1.6 | 3.5 | 2.2 | 5.1 |
| CSHA | Phospholipids (RBC or plasma) | 0 | 0.22 | 0.31 | 2.69 | 1.3 | 2.69 | 1.98 | 5.14 | 2.36 | 6.06 |
| FHS |  | 0.11 | 0.27 | 0.36 | 0.98 | 2.18 | 3.12 | 3.02 | 6.25 | 3.46 | 7.13 |
| WHIMS |  | 0.09 | 0.25 | 0.33 | 1.17 | 1.96 | 3.04 | 2.83 | 6.35 | 3.34 | 7.32 |
| AGES-R |  | 0.15 | 0.32 | 1.31 | 5.19 | 0.92 | 1.43 | 4.5 | 8.39 | 5.94 | 13.5 |
| CHS |  | 0.09 | 0.21 | 0.3 | 0.93 | 0.64 | 1.04 | 1.96 | 4.38 | 2.4 | 5.19 |
| EPIC-Norfolk |  | 0.13 | 0.34 | 0.58 | 2.09 | 0.97 | 1.89 | 3.32 | 7.28 | 4.09 | 9.22 |
| MCCS |  | 0.09 | 0.27 | 0.57 | 1.62 | 0.93 | 1.54 | 2.78 | 5.3 | 3.57 | 6.67 |
| MESA |  | 0.13 | 0.21 | 0.52 | 1.08 | 0.37 | 1.08 | 3.05 | 5.14 | 3.64 | 6.18 |
| MetSIM |  | 0.19 | 0.49 | 1.2 | 3.7 | 1.15 | 1.67 | 3.81 | 7.61 | 5.27 | 11.21 |
| HPFS |  | 0.11 | 0.27 | 0.25 | 0.80 | 1.44 | 2.44 | 2.17 | 5.16 | 2.50 | 5.85 |
| NHS |  | 0.08 | 0.20 | 0.24 | 0.61 | 1.07 | 1.93 | 1.83 | 4.15 | 2.14 | 4.69 |
|  |  |  |  |  |  |  |  |  |  |  |  |
| Summary |  | ALA | | EPA | | DPA | | DHA | | EPA+DHA | |
| Percentiles |  | **10^th^** | **90^th^** | **10^th^** | **90th** | **10th** | **90th** | **10^th^** | **90^th^** | **10^th^** | **90^th^** |
| **Mean – Cholesteryl esters** | | **0.56** | **1** | **0.92** | **2.68** | **NA** | **NA** | **0.54** | **1.1** | **1.52** | **4.29** |
| **Mean - Plasma** | | **0.43** | **0.95** | **0.61** | **2.27** | **0.43** | **0.82** | **1.87** | **4.13** | **2.58** | **6.25** |
| **Mean – PL** | | **0.11** | **0.28** | **0.54** | **1.90** | **1.18** | **1.99** | **2.84** | **5.92** | **3.52** | **7.55** |

† All abbreviations shown in Table 1.

**Table S5.** I^2^ and 2-sided p-value for Q-test of heterogeneity for mortality outcomes in Table 2.

| Fatty acid | All-cause | CVD | Cancer | Other |
| --- | --- | --- | --- | --- |
| ALA | 25.11% p=0.16 | 39.88% p=0.05 | 0%  p=0.65 | 18.86% p=0.24 |
| EPA | 40.93% p=0.04 | 0%  p=0.81 | 18.28% p=0.24 | 15.36% p=0.28 |
| DPA | 50.19% p=0.01 | 0%  p=0.47 | 33.46% p=0.11 | 43.29% p=0.05 |
| DHA | 71.06% p=0.00 | 56.23% p=0.003 | 0%  p=0.69 | 46.53% p=0.02 |
| EPA+DHA | 63.01% p=0.0002 | 42.29% p=0.04 | 0%  p=0.80 | 38.28% p=0.07 |

Abbreviations: ALA, alpha-linolenic acid; CI, confidence interval; CVD, cardiovascular disease; DHA, docosahexaenoic acid; DPA, docosapentaenoic acid; EPA, eicosapentaenoic acid.

**Table S6.** Exploration of potential heterogeneity in relationships of n-3 PUFA biomarkers with mortality outcomes by lipid compartment [Hazard ratios (95% CI)]

| Fatty acid | Compartment | All cause (17 cohorts) | CVD (15 cohorts) | Cancer (15 cohorts) | Other (14 cohorts) |
| --- | --- | --- | --- | --- | --- |
| ALA | Phospholipid (RBC/plasma) | 1.00 (0.97,1.04) | 0.99 (0.93,1.06) | 1.05 (0.98,1.11) | 1.01 (0.96,1.06) |
|  | Plasma | 0.89 (0.81,0.97) | 1.07 (0.91,1.25) | 0.86 (0.70,1.05) | 0.88 (0.76,1.02) |
|  | Cholesteryl Ester | 1.00 (0.91,1.10) | 1.08 (0.93,1.26) | 0.93 (0.79,1.11) | 0.96 (0.81,1.15) |
|  | P-value for difference | 0.05 | 0.45 | 0.10 | 0.22 |
| EPA | Phospholipid (RBC/plasma) | 0.89 (0.86,0.93) | 0.88 (0.81,0.95) | 0.89 (0.82,0.96) | 0.91 (0.85,0.97) |
|  | Plasma | 0.92 (0.87,0.99) | 0.90 (0.78,1.04) | 0.95 (0.83,1.09) | 0.94 (0.85,1.03) |
|  | Cholesteryl Ester | 0.93 (0.84,1.03) | 0.88 (0.73,1.05) | 0.95 (0.78,1.14) | 0.94 (0.77,1.15) |
|  | P-value for difference | 0.62 | 0.95 | 0.60 | 0.86 |
| DPA | Phospholipid (RBC/plasma) | 0.89 (0.85,0.93) | 0.91 (0.84,0.99) | 0.86 (0.79,0.94) | 0.90 (0.84,0.97) |
|  | Plasma | 0.76 (0.67,0.86) | 0.94 (0.73,1.20) | 0.93 (0.76,1.14) | 0.66 (0.52,0.84) |
|  | Cholesteryl Ester | NA | NA | NA | NA |
|  | P-value for difference | 0.02 | 0.82 | 0.52 | 0.014 |
| DHA | Phospholipid (RBC/plasma) | 0.86 (0.83,0.91) | 0.83 (0.76,0.90) | 0.92 (0.84,1.01) | 0.88 (0.82,0.94) |
|  | Plasma | 0.94 (0.85,1.04) | 1.05 (0.86,1.30) | 0.93 (0.76,1.16) | 0.87 (0.73,1.03) |
|  | Cholesteryl Ester | 0.94 (0.85,1.04) | 0.84 (0.70,1.01) | 0.96 (0.80,1.15) | 1.04 (0.88,1.24) |
|  | P-value for difference | 0.17 | 0.11 | 0.91 | 0.18 |
| EPA+DHA | Phospholipid (RBC/plasma) | 0.86 (0.83,0.91) | 0.81 (0.74,0.89) | 0.87 (0.80,0.96) | 0.87 (0.80,0.93) |
|  | Plasma | 0.94 (0.85,1.04) | 1.00 (0.83,1.21) | 0.92 (0.75,1.12) | 0.87 (0.74,1.01) |
|  | Cholesteryl Ester | 0.94 (0.85,1.04) | 0.86 (0.71,1.03) | 0.95 (0.78,1.15) | 0.96 (0.79,1.17) |
|  | P-value for difference | 0.14 | 0.13 | 0.72 | 0.63 |

Abbreviations: ALA, alpha-linolenic acid; CI, confidence interval; CVD, cardiovascular disease; DHA, docosahexaenoic acid; DPA, docosapentaenoic acid; EPA, eicosapentaenoic acid. Number of cohorts reporting fatty acid data by compartment: RBC/PL, 11; CE, 2; Plasma, 4.

**Table S7. Associations of n-3 fatty acids with all-cause mortality stratified by age, sex, race/ethnicity, or fish oil use.**

|  | **Age (years)** | | **Sex** | | **Race** | | **Fish oil use** | |
| --- | --- | --- | --- | --- | --- | --- | --- | --- |
|  | Category | HR (95% CI) | Category | HR (95% CI) | Category | HR (95% CI) | Category | HR (95% CI) |
| **ALA** | ≥60 (n=28,716) | 0.99 (0.96,1.02) | Female (n=23,359) | 0.99 (0.92,1.06) | Non-white (n=5,692) | 0.97 (0.87,1.07) | Fish oil^2^ (n=1,112) | 1.11 (0.98,1.24) |
|  | <60 (n=13,750) | 0.99 (0.92,1.06) | Male (n=19,107) | 1.00 (0.96,1.04) | White (n=36,774) | 0.99 (0.96,1.02) | No Fish oil (n=41,354) | 0.99 (0.96,1.02) |
|  | P-values  unadjusted (adjusted^1^) | 0.99 (0.98) |  | 0.36 (0.50) |  | 0.65 (0.39) |  | 0.06 (0.08) |
| **EPA** | ≥60 (n=28,716) | 0.90 (0.87,0.93) | Female (n=23,359) | 0.87 (0.82,0.91) | Non-white (n=5,692) | 0.93 (0.87,1.00) | Fish oil^2^ (n=1,112) | 0.70 (0.52,0.94) |
|  | <60 (n=13,750) | 0.98 (0.90,1.06) | Male (n=19,107) | 0.91 (0.87,0.95) | White (n=36,774) | 0.89 (0.86,0.92) | No Fish oil (n=41,354) | 0.90 (0.87,0.93) |
|  | P-values  unadjusted (adjusted^1^) | 0.06 (0.03) |  | 0.10 (0.10) |  | 0.26 (0.22) |  | 0.09 (0.08) |
| **DPA^3^** | ≥60 (n=24,140) | 0.89 (0.85,0.92) | Female (n=20,649) | 0.88 (0.83,0.93) | Non-white (n=5,692) | 0.71 (0.59,1.85) | Fish oil^2^ (n=1,112) | 0.99 (0.85,1.16) |
|  | <60 (n=10,955) | 0.92 (0.80,1.06) | Male (n=14,446) | 0.89 (0.84,0.94) | White (n=29,403) | 0.89 (0.85,0.92) | No Fish oil (n=33,983) | 0.88 (0.84,0.91) |
|  | P-values  unadjusted (adjusted^1^) | 0.63 (0.64) |  | 0.76 (0.96) |  | 0.02 (0.02) |  | 0.14 (0.21) |
| **DHA** | ≥60 (n=28,716) | 0.88 (0.85,0.91) | Female (n=23,359) | 0.85 (0.81,0.90) | Non-white (n=5,692) | 0.91 (0.81,1.03) | Fish oil^2^ (n=1,112) | 0.94 (0.77,1.15) |
|  | <60 (n=13,750) | 0.97 (0.89,1.06) | Male (n=19,107) | 0.87 (0.83,0.92) | White (n=36,774) | 0.87 (0.84,0.90) | No Fish oil (n=41,354) | 0.87 (0.84,0.91) |
|  | P-values  unadjusted (adjusted^1^) | 0.04 (0.05) |  | 0.60 (0.80) |  | 0.49 (0.39) |  | 0.45 (0.61) |
| **EPA+DHA** | ≥60 (n=28,716) | 0.86 (0.83,0.89) | Female (n=23,359) | 0.83 (0.78,0.88) | Non-white (n=5,692) | 0.91 (0.81,1.01) | Fish oil^2^ (n=1,112) | 0.80 (0.62,1.04) |
|  | <60 (n=13,750) | 0.96 (0.88,1.04) | Male (n=19,107) | 0.86 (0.82,0.91) | White (n=36,774) | 0.85 (0.82,0.89) | No Fish oil (n=41,354) | 0.86 (0.83,0.89) |
|  | P-values  unadjusted (adjusted^1^) | 0.02 (0.03) |  | 0.31 (0.42) |  | 0.28 (0.24) |  | 0.61 (0.54) |

^1^ p-value for difference adjusted for fatty acid compartment.
^2^ Only individuals in the AGES-R cohort taking fish oil were included here. All other cohorts excluded participants taking fish oil per analytic protocol.
^3^Sample sizes for DPA are smaller since the 60YO, CCCC and ULSAM50 cohorts did not measure DPA. Hazard ratios (HRs) and CIs were expressed per a cohort-specific range of the 10^th^ (reference) to the 90^th^ percentile of each fatty acid.

**Figure S1. Omega-3 and Omega-6 fatty acid levels by cohort and lipid class**. Mean values are represented by a colored diamond, and the SD is represented by the horizontal bars traversing them. Abbreviations of fatty acids: ALA, alpha-linolenic acid; EPA, eicosapentaenoic acid; DPA, docosapentaenoic acid; DHA, docosahexaenoic acid; LA, linoleic acid; AA, arachidonic acid; CE, cholesteryl esters; PL, phospholipids; RBC, red blood cell. Of cohorts, 60YO, Stockholm cohort of 60-year olds; AGESR: Age, Genes, Environment Susceptibility Study (Reykjavik); CCCC, Chin-Shan Community Cardiovascular Cohort Study; CHS, Cardiovascular Health Study; CSHA, Canadian Study of Health and Aging; EPIC-Norfolk, European Prospective Investigation into Cancer, Norfolk UK; FHS, Framingham Heart Study; HS, Hisayama Study; HPFS, Health Professionals Follow-up Study; KIHD, Kuopio Ischaemic Heart Disease; MCCS, Melbourne Collaborative Cohort Study; MESA, Multi-Ethnic Study of Atherosclerosis; MetSIM, Metabolic Syndrome in Men Study; NHS, Nurses Health Study; Three C, Three City Study; ULSAM, Uppsala Longitudinal Study of Adult Men; WHIMS, Women’s Health Initiative Memory Study.


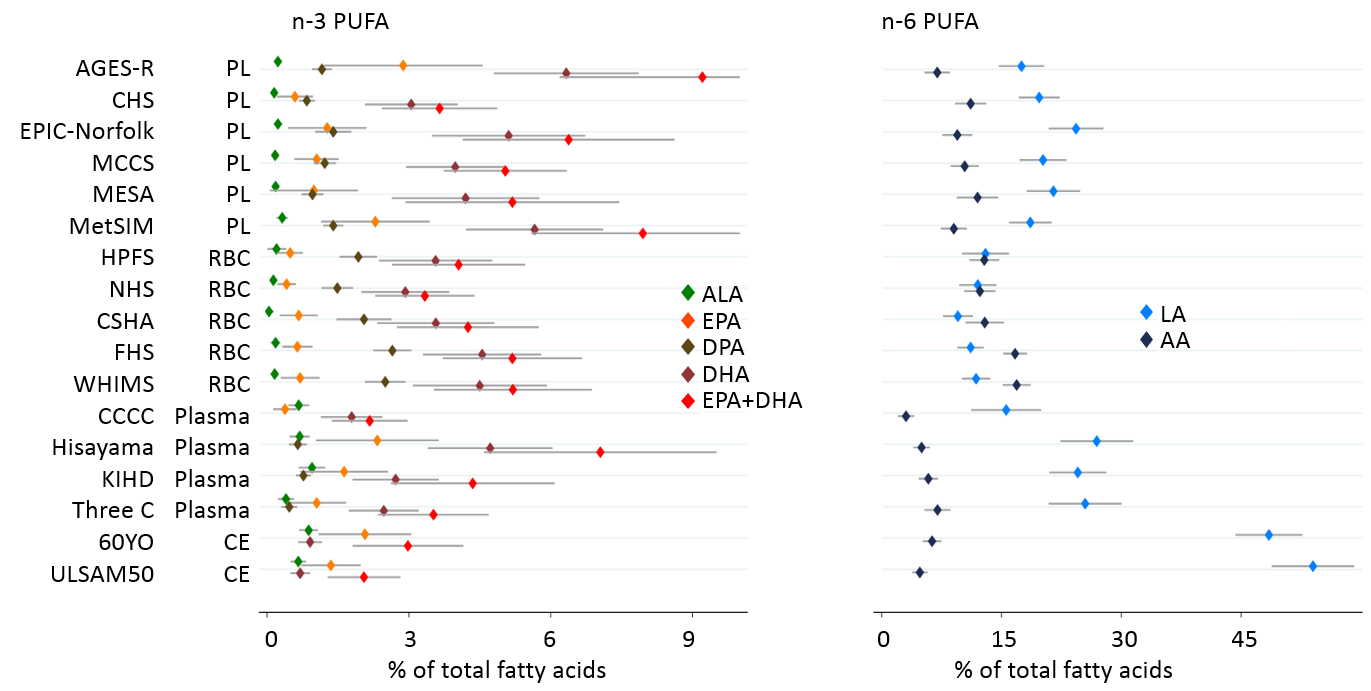


**Figure S2.** Associations of circulating ALA levels with all-cause mortality: non-linear dose-response meta-analysis in the Fatty Acids and Outcomes Research Consortium. Hazard ratios and cohort-specific quantiles are presented in the vertical and horizontal axis, respectively. The best estimates and their confidence intervals are presented as black lines and grey-shaded areas, respectively. The 10^th^ percentile was selected as a reference level. As shown in corresponding Figure 2, potential non-linearity was identified for EPA (p=0.0004) and not for the others (p>0.05) using the 2-sided testing method of Orsini N, et al. [Meta-Analysis for Linear and Nonlinear Dose-Response Relations: Examples, an Evaluation of Approximations, and Software. American Journal of Epidemiology. 2011;175(1):66-73] without correction for multiple testing. In particular, the p-value for non-linearity for ALA = 0.30. All HRs are adjusted for age, sex, race, field center, body-mass index, education, occupation, marital status, smoking, physical activity, alcohol intake, prevalent diabetes, hypertension, and dyslipidemia, self-reported general health, and the sum of circulating n-6 PUFA (linoleic plus arachidonic acids).


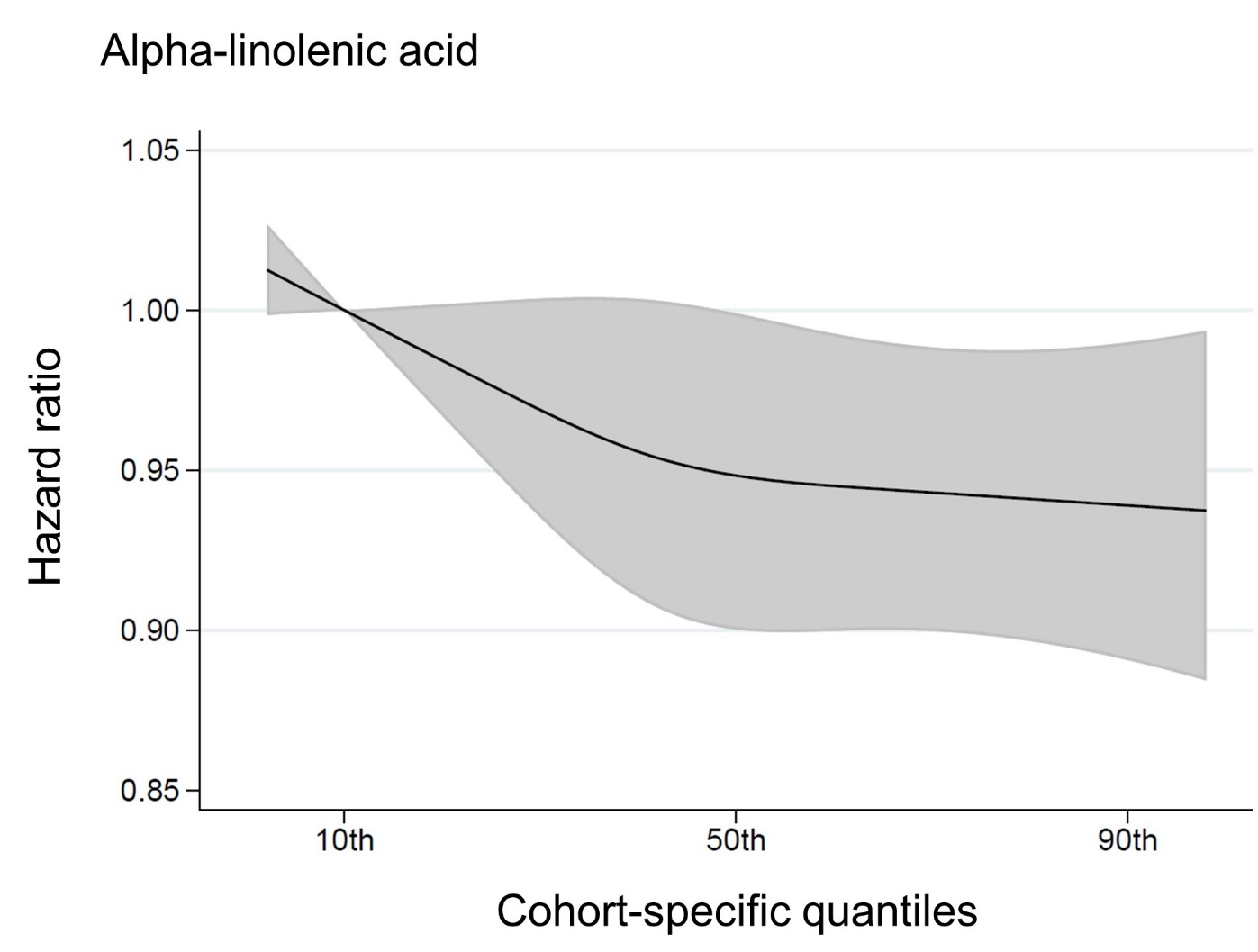

Supplement: Supplementary file 1 — Supplementary Information [file 41467_2021_22370_MOESM1_ESM.docx]
