# Supplementary material for: Blood n-3 fatty acid levels and total and cause-specific mortality from 17 prospective studies
Source: Nat Commun. 2021 Apr 22;12:2329. doi: 10.1038/s41467-021-22370-2 (PMC8062567; doi:10.1038/s41467-021-22370-2)
Supplement: Supplementary file 3 — Reporting Summary [file 41467_2021_22370_MOESM3_ESM.pdf]

nature research

Corresponding author(s): William S. Harris, PhD

Last updated by author(s): Mar 5, 2021

Reporting Summary

Nature Research wishes to improve the reproducibility of the work that we publish. This form provides structure for consistency and transparency in reporting. For further information on Nature Research policies, see our Editorial Policies and the Editorial Policy Checklist.

Statistics

For all statistical analyses, confirm that the following items are present in the figure legend, table legend, main text, or Methods section.

n/a	Confirmed	
☐	☒	The exact sample size (n) for each experimental group/condition, given as a discrete number and unit of measurement
☐	☒	A statement on whether measurements were taken from distinct samples or whether the same sample was measured repeatedly
☐	☒	The statistical test(s) used AND whether they are one- or two-sided<br>*Only common tests should be described solely by name; describe more complex techniques in the Methods section.*
☐	☒	A description of all covariates tested
☐	☒	A description of any assumptions or corrections, such as tests of normality and adjustment for multiple comparisons
☐	☒	A full description of the statistical parameters including central tendency (e.g. means) or other basic estimates (e.g. regression coefficient) AND variation (e.g. standard deviation) or associated estimates of uncertainty (e.g. confidence intervals)
☐	☒	For null hypothesis testing, the test statistic (e.g. F, t, r) with confidence intervals, effect sizes, degrees of freedom and P value noted<br>*Give P values as exact values whenever suitable.*
☒	☐	For Bayesian analysis, information on the choice of priors and Markov chain Monte Carlo settings
☒	☐	For hierarchical and complex designs, identification of the appropriate level for tests and full reporting of outcomes
☐	☒	Estimates of effect sizes (e.g. Cohen's d, Pearson's r), indicating how they were calculated

Our web collection on statistics for biologists contains articles on many of the points above.

Software and code

Policy information about availability of computer code

Data collection	Each of the 17 cohorts participating used its own system for collecting raw data.
Data analysis	R version 3 was used for the meta-analysis, and Stata 15.1 was used for spline fitting.

For manuscripts utilizing custom algorithms or software that are central to the research but not yet described in published literature, software must be made available to editors and reviewers. We strongly encourage code deposition in a community repository (e.g. GitHub). See the Nature Research guidelines for submitting code & software for further information.

Data

Policy information about availability of data

All manuscripts must include a data availability statement. This statement should provide the following information, where applicable:

- Accession codes, unique identifiers, or web links for publicly available datasets
- A list of figures that have associated raw data
- A description of any restrictions on data availability

Policies for data-sharing vary between the cohorts depending on their original human subjects approvals and existing procedures. For approved data-sharing requests, types of data that may be shared can include demographics, exposures, covariates, and outcomes. Please contact each individual principal investigator for cohort-specific data requests (See Supplementary Table 1).

Field-specific reporting

Please select the one below that is the best fit for your research. If you are not sure, read the appropriate sections before making your selection.

- [x] Life sciences
- [ ] Behavioural & social sciences
- [ ] Ecological, evolutionary & environmental sciences

For a reference copy of the document with all sections, see nature.com/documents/nr-reporting-summary-flat.pdf

Life sciences study design

All studies must disclose on these points even when the disclosure is negative.

Sample size	17 separate prospective cohort studies were pooled in this analysis. Each had its own n (as shown in Table 1). The total sample size was 42,466. The authors of this meta-analysis were not involved with selecting those original sample sizes.
Data exclusions	(a) were >18 years old, (b) had no major medical diagnoses (prior myocardial infarction, prior stroke, severe active cancer, severe renal disease, severe liver or lung disease), (c) were not taking supplemental fish oil and (d) did not die within a year of baseline.
Replication	We did a 'drop one' sensitivity analysis to confirm that no one study drove the overall findings.
Randomization	Not a randomized trial
Blinding	Observational trial

Reporting for specific materials, systems and methods

We require information from authors about some types of materials, experimental systems and methods used in many studies. Here, indicate whether each material, system or method listed is relevant to your study. If you are not sure if a list item applies to your research, read the appropriate section before selecting a response.

Materials & experimental systems

n/a	Involved in the study	
x		Antibodies
x		Eukaryotic cell lines
x		Palaeontology and archaeology
x		Animals and other organisms
	x	Human research participants
x		Clinical data
x		Dual use research of concern

Methods

n/a	Involved in the study	
x		ChIP-seq
x		Flow cytometry
x		MRI-based neuroimaging

Human research participants

Policy information about studies involving human research participants

Population characteristics	There were 42,466 individuals included, and 15,720 (35%) died during follow-up. At baseline, the average age was 64 years (range of mean ages across cohorts was 50-81 years), 55% were women (range of 0 to 100% across cohorts) and the median follow-up time was 16 years (range of 5 to 32 years across cohorts). Whites constituted 86% of the sample.
Recruitment	This is described in Supplemental Table 1 for each of the 17 participating cohorts
Ethics oversight	Relevant IRBs for each cohort now listed in the updated manuscript

Note that full information on the approval of the study protocol must also be provided in the manuscript.
